# Supplementary material for: Physiology and ecology combine to determine host and vector importance for Ross River virus
Source: eLife. 2021 Aug 20;10:e67018. doi: 10.7554/eLife.67018 (PMC8457839; doi:10.7554/eLife.67018)
Supplement: Supplementary file 1. [file elife-67018-supp1.docx]

**Table S1: Summary of host data.** “Physiological competence data” is the data we extracted from published literature on experimental infections of host species with RRV, which we used to model continuous host titer profiles (Figure S_m_1). For further detail on variation in detected maxima and durations see Figure S_m_1 and each listed publication. “Brisbane-specific `ecological` data” includes both the estimated densities of each host species in Brisbane and the percent of each host species detected seropositive for RRV. All sources are cited in the main text’s references section.

| **Host species** | **Physiological competence data** | | | | **Brisbane-specific “ecological” data** | | | |
| --- | --- | --- | --- | --- | --- | --- | --- | --- |
|  | Titer maximum (unit) | Titer duration range (days) | Percentage viremic range (total tested) | Source | Density (per km^2^) | Source | Seroprevalence (%) (total tested) | Source |
| Humans | 2.5-4.9 (LD50) | - | 20-56 (102) | Rosen et al. 1981 | 917 | ABS census (2018) | 13.8 (355) | Faddy et al. 2015 |
| Brushtail possum | 8.5 (CCID50) | 3 | 33 (10) | Boyd & Kay 2001 | 22 | Environmental Protection Agency (2020) | 53.8 (327) | Skinner, Rudd et. al 2020 |
| Agile wallaby | 5.6 (SMIC50) | 3.4 | 78 (9) | Kay et al. 1986 | 7 | Environmental Protection Agency (2020) | 43.9 (2632) | Potter et al. 2014 |
| Grey kangaroo | 4.6 (SMIC50) | 6 | 100 (3) | Kay et al. 1986 |  |  |  |  |
| Horse | 6.3 (SMIC50) | 4 | 10 (11) | Kay et al. 1986 | 0.2 | Ward et al. 1996 | 93.9 (82) | Skinner, Rudd et al. 2020 |
| Sheep | 4.5-6.5 (LD50) | 2-5 | 100 (20) | Kay et al. 1986; Whitehead 1969; Spradbrow 1973 | 0.4 | Meat and Livestock Australia (2020) | 1.1 (36) | Doherty et al. 1966 |
| Cow | 2.3 (SMIC50) | 2 | 16 (6) | Kay et al. 1986 | 14 | Meat and Livestock Australia (2020) | 3.6 (187) | Vale et al. 1991 |
| Cat | 0 (CCID50) | 0 | 0 (10) | Boyd & Kay 2002 | 138 | Animal Medicine Australia (2019) | 1.4 (433) | Boyd & Kay 2002 |
| Dog | 0 (CCID50) | 0 | 0 (10) | Boyd & Kay 2002 | 187 | Animal Medicine Australia (2019) | 23.7 (454) | Boyd & Kay 2002 |
| Flying foxes | 2.2 (TCID50) | 4 | 33 (10) | Ryan et al. 1997 | 19 | Queensland Government (2020) | 17.2 (38) | Skinner, Rudd et al. 2020 |
| Rat | 4 (LD50) | 3 | 100 (4) | Whitehead 1969 | 0.2 | Raw data from Skinner et al. 2020 | 0.2 (150) | Doherty et al. 1966 |
| Rabbit | 3.1-4.7 (SMIC50)  (LD50) | 2-2.3 | 0-67 (13) | Kay et al. 1986; Whitehead 1969 | 0.2 | Raw data from Skinner et al. 2020 | 0 (1) | Marshall et al. 1980 |
| Chicken | 2.8-5 (SMIC50) | 2.9-5 | 18.75-95 (52) | Kay et al. 1986; Whitehead 1969 | 73 | BirdLife Australia (2020) | 28.9 (97) | Skinner, Rudd et al 2020 |
| Black duck | 1.8 (SMIC50) | 4 | 67 (3) | Kay et al. 1986 |  |  |  |  |
| Little corella | 2.3 (SMIC50) | 2.1 | 50 (12) | Kay et al. 1986 |  |  |  |  |
