# Supplementary material for: Physiology and ecology combine to determine host and vector importance for Ross River virus
Source: eLife. 2021 Aug 20;10:e67018. doi: 10.7554/eLife.67018 (PMC8457839; doi:10.7554/eLife.67018)
Supplement: Supplementary file 2. [file elife-67018-supp2.docx]

**Table S2: Summary of vector data.** “Physiological competence data” is the data we extracted from published literature on experimental infections of mosquito species with RRV, which we used to model continuous mosquito infection (Figure S_m_3) and transmission probability (Figure S_m_5) curves. For further detail on the variation in mosquito infection and transmission probabilities see Figure S_m_3, Figure S_m_5, and each listed publication. “Brisbane-specific `ecological` data” includes both the estimated abundances of each host species in Brisbane and the total number of individual mosquitoes of each species included in the blood meal analyses; for a breakdown of the percent of each host species’ blood detected in each mosquito species see Table S4. In the “Source” column “Brisbane-specific `ecological` data”, all three citations were used fro weekly abundance while only Kay et al. 2007 and Jansen et al. 2009 were used for blood meal data. All sources are listed in the main text’s references section.

| **Vector species** | **Physiological competence data** | | | | **Brisbane-specific “ecological” data** | | |
| --- | --- | --- | --- | --- | --- | --- | --- |
|  | Dose range | Infection % range (total infected) | Transmission % range (total tested) | Source | Average weekly abundance | Total number blood fed in Brisbane | Source |
| *Aedes notoscriptus* | 3.5-7.7 (CCID50, TCID50) | 0-100 (774) | 0-100 (217) | Doggett and Russell 1997; Ryan, Do & Kay 2000; Watson & Kay 1998 | 761 | 181 | Skinner et al. 2020; Jansen et al. 2009; Kay et al. 2007 |
| *Aedes procax* | 5.7-7.7 (CCID50) | 30-100 (259) | 0-93 (102) | Ryan, Do & Kay 2000 | 6184 | 29 | Skinner et al. 2020; Jansen et al. 2009; Kay et al. 2007 |
| *Aedes vigilax* | 2.9-9 (CCID50, TCID50, SMIC) | 0-100 (2632) | 0-100 (351) | Jefferey et al. 2002; Jefferey et al. 2006; Jennings & Kay 1999; Kay & Jennings 2002; Ramirez et al. 2018; Ryan, Do & Kay 2000; Watson & Kay 1998 | 7199 | 153 | Skinner et al. 2020; Jansen et al. 2009; Kay et al. 2007 |
| *Coquillettidia linealis* | 4.2-7 (CCID50) | 32-100 (301) | 4-92 (150) | Jefferey et al. 2002 | 3151 | 6 | Skinner et al. 2020; Jansen et al. 2009; Kay et al. 2007 |
| *Culex annulirostris* | 4.7-8.5 (CCID50, TCID50) | 0-83 (508) | 8-46 (42) | Doggett, Klowden & Russell 2001; Ryan, Do & Kay 2000 | 10998 | 508 | Skinner et al. 2020; Jansen et al. 2009; Kay et al. 2007 |
| *Culex quinquefasciatus* | 5.2-8.1 (LD50) | 0-22 (484) | 0 (0) | Kay, Fanning & Carley 1982 | 131 | 27 | Skinner et al. 2020; Jansen et al. 2009; Kay et al. 2007 |
| *Culex sitiens* | 5.7-9.3 (CCID50, TCID50) | 0-65 (374) | 10-30 (84) | Fanning, Mottram & Kay 1992 | 185 | 17 | Skinner et al. 2020; Jansen et al. 2009; Kay et al. 2007 |
| *Mansonia uniformis* | 7.7 (CCID50) | 80 (5) | 60 (5) | Ryan, Do & Kay 2000 | 76 | 1 | Skinner et al. 2020; Jansen et al. 2009 |
| *Verrallina funerea* | 4.7-7.7 (CCID50, TCID50) | 0-100 (532) | 25-58 (55) | Jefferey et al. 2006; Ryan, Do & Kay 2000 | 45 | 1 | Skinner et al. 2020; Jansen et al. 2009 |
