# Supplementary material for: Physiology and ecology combine to determine host and vector importance for Ross River virus
Source: eLife. 2021 Aug 20;10:e67018. doi: 10.7554/eLife.67018 (PMC8457839; doi:10.7554/eLife.67018)
Supplement: Supplementary file 3. [file elife-67018-supp3.docx]

**Table S3: Summary of mosquito blood meal data.** The number of blood meals detected in each mosquito species considered in our transmission model from each host species included in the model. The total number of blood meals for a given mosquito species (row sums) may be slightly smaller than the sample size listed in Table S3 because of bites on other species or blood meals listed as “multiple species” which we discard for this analysis (e.g., *Aedes vigilax* has a total sample size of 150 here as opposed to 153 total blood meals as listed in Table S3). See *Methods: Mosquito feeding behavior* and *Supplemental Methods: Mosquito vectors: feeding behavior* for details on how these data were used to model mosquito feeding preferences conditioned on host abundance.

|  | Rat | Macropod | Sheep | Rabbit | Humman | Possum | Bird | Flying Fox | Horse | Cattle | Dog | Cat |
| --- | --- | --- | --- | --- | --- | --- | --- | --- | --- | --- | --- | --- |
| *Coquillettidia linealis* | 0 | 0 | 0 | 0 | 0 | 0 | 2 | 0 | 4 | 0 | 0 | 0 |
| *Aedes procax* | 0 | 0 | 0 | 0 | 3 | 0 | 8 | 0 | 1 | 0 | 17 | 0 |
| *Verrallina funerea* | 0 | 0 | 0 | 0 | 1 | 0 | 0 | 16 | 0 | 0 | 0 | 0 |
| *Aedes vigilax* | 2 | 2 | 0 | 0 | 27 | 12 | 40 | 1 | 20 | 0 | 44 | 2 |
| *Mansonia uniformis* | 0 | 0 | 0 | 0 | 0 | 0 | 0 | 0 | 0 | 0 | 1 | 0 |
| *Culex annulirostris* | 3 | 21 | 0 | 1 | 58 | 57 | 54 | 1 | 102 | 3 | 200 | 7 |
| *Aedes notoscriptus* | 0 | 5 | 0 | 0 | 35 | 42 | 29 | 4 | 0 | 0 | 63 | 3 |
| *Culex quinquefasciatus* | 0 | 0 | 0 | 0 | 0 | 1 | 9 | 0 | 11 | 0 | 3 | 3 |
| *Culex sitiens* | 0 | 0 | 0 | 0 | 2 | 0 | 11 | 0 | 2 | 0 | 2 | 0 |
